# Lower average cortical bone thickness predicts cemented fixation in short-stem reverse shoulder arthroplasty

**DOI:** 10.1186/s13018-025-06606-1

**Published:** 2026-01-19

**Authors:** Felix Hochberger, Thilo Lehmeyer, Weinan Zeng, Maximilian Rudert, Kilian List

**Affiliations:** 1 Department of Orthopaedic Surgery, Julius-Maximilians University Wuerzburg, Koenig-Ludwig-Haus, Brettreichstrasse 11, 97074 Wuerzburg, Germany; 2 Department of Orthopedic Surgery and Orthopedic Research Institution, West China Hospital, Sichuan University, Chengdu, 610041 People’s Republic of China

**Keywords:** Reverse shoulder arthroplasty, Humeral component fixation, Cortical bone thickness, Radiographic assessment, Bone quality, Patient-related factors

## Abstract

**Purpose:**

This study aimed to identify radiographic and demographic predictors influencing the intraoperative decision for cemented versus cementless humeral fixation in patients ≤ 80 years undergoing short-stem reverse shoulder arthroplasty (RSA).

**Methods:**

A retrospective analysis was conducted on RSA cases between 02/2019 and 10/2024. Patients ≤ 80 years were stratified into Group A (cementless fixation; *n* = 209) or Group B (cemented fixation; *n* = 58) based on intraoperative assessment of trial stem stability and bone quality. Preoperative variables included age, sex, body mass index (BMI), American Society of Anesthesiologists score (ASA), diagnosis, and radiographic parameters such as cortical bone thickness gauge (CBTg), average cortical thickness (CBTavg), acromiohumeral distance (AHD), and Hamada and Walch classifications. Multivariate logistic regression including age, sex, CBTavg, CBTg, and diagnosis was performed to identify independent predictors of cemented fixation.

**Results:**

Patients in the cemented group were significantly older (74.0 ± 4.2 vs. 70.0 ± 6.1 years; *p* < 0.05) and more often female (81% vs. 61%; *p* < 0.05). Cortical bone measurements were significantly lower in the cemented group (CBTg: 0.25 ± 0.06 vs. 0.27 ± 0.06; CBTavg: 5.15 mm ± 1.23 mm vs. 6.42 mm ± 1.43 mm; both *p* < 0.05). Multivariate analysis identified increasing age (OR: 1.1; *p* < 0.05), female sex (OR: 2.8; *p* < 0.05), and reduced CBTavg (OR: 0.6; *p* < 0.05) as independent predictors of cemented fixation. Other variables such as BMI, ASA score, AHD, and CBTg did not show significant associations with fixation type.

**Conclusion:**

Among all evaluated factors, lower CBTavg was the strongest independent predictor for the use of cemented humeral fixation in short-stem RSA. Its integration into preoperative planning may assist surgical decision-making and improve consistency in fixation strategy.

**Study design:**

Level IV; retrospective case series.

**Supplementary Information:**

The online version contains supplementary material available at 10.1186/s13018-025-06606-1.

## Introduction

Reverse shoulder arthroplasty (RSA) has become a widely accepted treatment for various shoulder pathologies, including cuff tear arthropathy (CTA), osteoarthritis (OA), and irreparable rotator cuff tears [1]. A critical intraoperative decision in RSA is the choice between cemented and uncemented fixation of the humeral component, which may influence implant stability, complication rates, and long-term outcomes. Previous studies have demonstrated comparable functional and clinical results between both fixation types, yet each presents distinct advantages and potential drawbacks [2–5]. Cemented stems provide immediate primary stability but may increase the risk of infection, thermal bone injury, and technical challenges during revision [6]. In contrast, uncemented stems promote osseointegration and avoid cement-related complications but have been associated with a higher incidence of early migration and radiolucent lines [7].

Despite growing evidence comparing fixation techniques, the preoperative factors influencing the intraoperative choice of fixation strategy remain insufficiently understood. Preliminary data suggest that lower cortical bone thickness (CBTavg/CBTg) of the humeral diaphysis may predispose to cemented fixation [8]. In addition, patient-specific characteristics such as age, sex, and underlying pathology have been proposed to affect fixation choice, although robust evidence supporting these associations is limited [9]. As fixation type is typically determined intraoperatively based on surgeon judgment, retrospective analyses are inherently prone to selection bias. While registry studies have reported differences in revision rates between cemented and uncemented stems [10], dedicated investigations systematically evaluating intraoperative fixation decisions based on radiographic and patient-related parameters remain lacking.

Recent advances include 3D CT-based bone density quantification [11] and machine-learning-driven classification of bone quality subgroups from patient-specific CT scans in RSA cohorts [12], which may complement traditional cortical thickness metrics and further refine preoperative fixation strategy. However, as CT imaging is not routinely available or standardized for all shoulder arthroplasty cases, radiographic cortical bone thickness assessment remains a well-established and scientifically validated method for estimating local bone quality [13].

The present study aims to analyze the influence of preoperative radiographic, patient-related, and surgery-specific factors on the intraoperative decision to use cemented versus uncemented fixation in RSA. By conducting a retrospective analysis of a large patient cohort, we seek to determine whether age, sex, average and gauge cortical bone thickness (CBTavg/CBTg) of the humeral diaphysis, and preoperative diagnosis significantly affect fixation strategy. The authors hypothesized that lower cortical bone thickness parameters (CBTavg and CBTg), higher body mass index (BMI), and female sex would be independently associated with an increased likelihood of cemented fixation. The findings of this study may contribute to a more refined individualized approach to humeral fixation in RSA, ultimately optimizing surgical decision-making and improving patient outcomes.

## Materials and methods

### Patient selection

This retrospective study screened patients who underwent short-stem reverse shoulder arthroplasty (RSA) at a single orthopaedic center between 02/2019 and 10/2024. Inclusion criteria comprised patients with complete preoperative imaging, detailed surgical documentation, and postoperative follow-up data. Patients older than 80 years were excluded to ensure a homogeneous cohort representative of elective primary short-stem RSA. At our institution, stem cementation is generally preferred in patients over 80 years to enhance primary stability and reduce the lifetime risk of revision due to aseptic loosening. To minimize this potential source of bias, the present analysis focused on patients aged ≤ 80 years. Moreover, patients with prior surgeries on the same shoulder before RSA, malignant or neuromuscular diseases, insufficient documentation, missing radiographic data as well as cases with intraoperative fissures or fractures requiring cemented stem fixation were excluded. Additionally, proximal humerus fractures were excluded because these cases are routinely treated with fracture-specific cemented stems at our institution, that differ fundamentally in geometry and fixation concept from the two short-stem designs evaluated in this study. Ethics approval at the author’s institution was obtained (Ref. 2024012901). An a priori power analysis (G*Power 3.1, Düsseldorf, Germany) was performed to determine the required sample size. A moderate-to-large effect size (Cohen’s d = 0.5) was assumed, reflecting the institutional observation that cementation is reserved for cases with markedly reduced bone quality or insufficient primary stability. With α = 0.05 and power 0.80, a minimum of 45 cases was required for the smaller cemented group. The final cohort (*n* = 267; 58 cemented) exceeded this threshold.

## Group allocation

Patients were assigned to two groups based on the fixation method for the humeral component, determined intraoperatively. Cemented fixation (Group B) was performed in cases where intraoperative stability testing, using a trial prosthesis stem, indicated inadequate primary stability of the humeral implant. Trial stem stability was assessed intraoperatively via manual torsional and axial testing. If visible or palpable notable motion was detected, cemented fixation was performed, consistent with methods recently described in the literature [14]. In contrast, cementless fixation (Group A) was chosen when stability testing confirmed sufficient bone-implant stability to allow for press-fit implantation without the need for cement. The decision-making process was predominantly guided by intraoperative findings. Preoperative patient-related and radiographic factors, as well as surgery-related factors were systematically analyzed.

## Surgical management and postoperative rehabilitation protocol

All RSA procedures were performed by a fellowship-trained, high-volume shoulder surgeon (K.L.) using a standardized deltopectoral approach. Preoperative assessment included radiographs and CT scans to evaluate glenoid morphology and humeral bone quality. Two short-stem reverse shoulder arthroplasty systems from the same manufacturer were used (Ascend Flex and Perform Humeral; Stryker, Kalamazoo, MI, USA), both sharing comparable fixation principles and surgical technique. Stem sizing and fixation followed the manufacturer’s standard protocols. Primary stability was assessed intraoperatively by manual testing of axial and rotational stability after trial stem insertion. Cemented fixation was chosen when notable micromotion was observed despite correct sizing, consistent with previously described methods [14]. In all other cases, metaphyseal press-fit fixation was applied. Implant selection was based on availability and the surgeon’s preference, ensuring a consistent fixation philosophy across all procedures.

Postoperative management followed a standardized rehabilitation protocol. The operated arm was immobilized in an abduction sling for the first 4 weeks, allowing early passive motion within a pain-free range. Active-assisted and active exercises were gradually introduced thereafter under physiotherapeutic supervision. Full range of motion and strengthening were permitted from 12 weeks postoperatively.

## Radiographic assessment

**Fig. 1:**
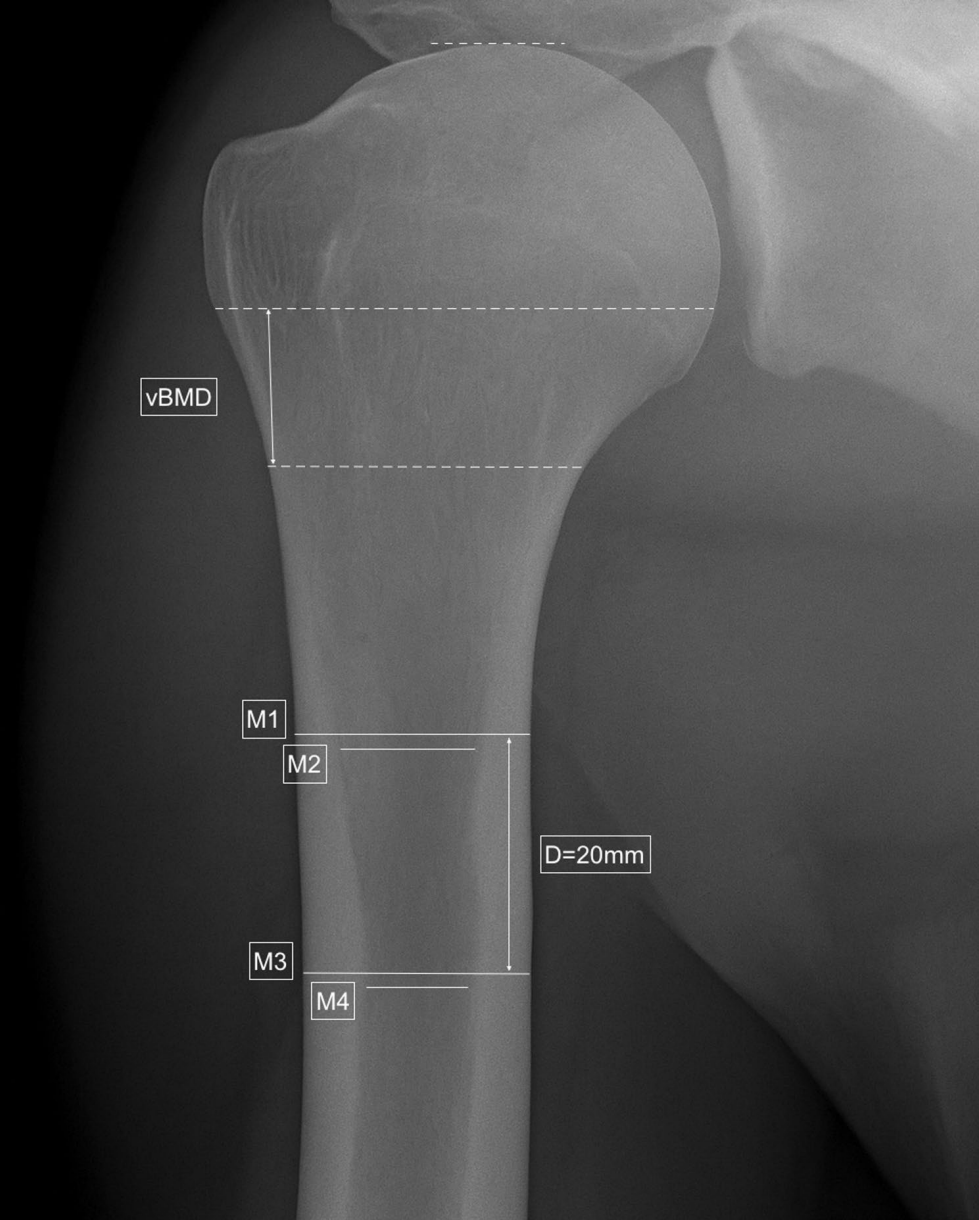
Schematic illustration of cortical bone thickness measurements (CBTg and CBTavg) on standardized anteroposterior radiographs. Measurements were taken at two diaphyseal levels (M1/M2 and M3/M4) separated by 20 mm. Outer (M1, M3) and inner (M2, M4) cortical diameters were used to calculate CBTg and CBTavg as described in the Methods. The figure serves for illustration only

**Fig. 2:**
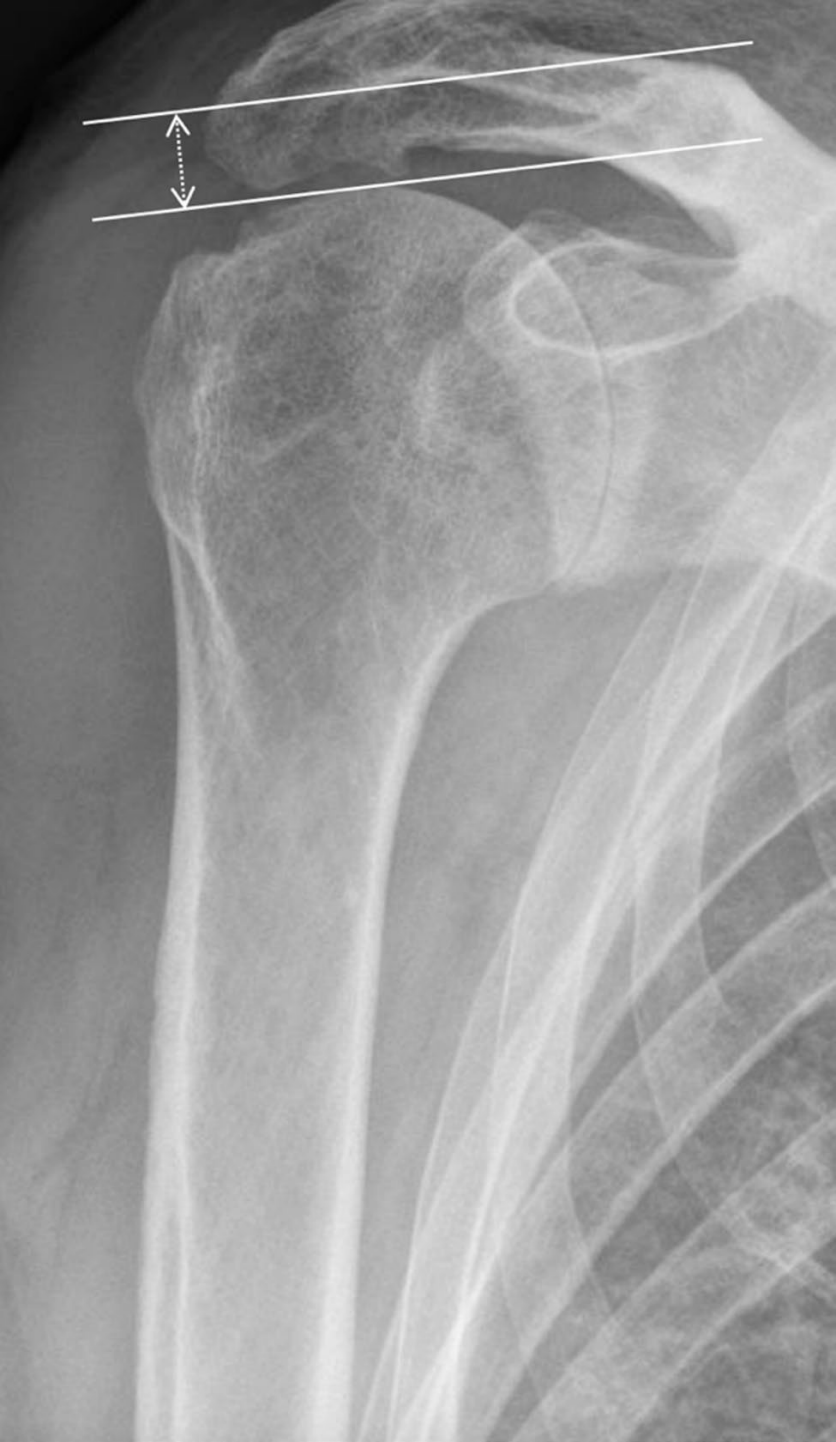
Measurement of the acromiohumeral distance (AHD) on a standardized anteroposterior radiograph, defined as the perpendicular distance between the undersurface of the lateral acromion and the superior border of the greater tuberosity

Preoperative imaging included standardized true anteroposterior, axial, and Y-view radiographs, complemented by computed tomography (CT) to assess glenoid morphology and humeral bone quality. The acromiohumeral distance (AHD) was measured as the perpendicular distance from the undersurface of the lateral acromion to the superior border of the greater tuberosity, serving as an indicator of superior humeral migration and rotator cuff integrity [15] (Fig. 2). Humeral bone quality was evaluated using the cortical bone thickness gauge (CBTg) and average cortical bone thickness (CBTavg) according to Schmidutz et al. [13]. Measurements were obtained at two standardized diaphyseal levels where the medial and lateral cortices ran parallel (M1/M2) and 20 mm distally (M3/M4) (Fig. 1). CBTg was calculated as the ratio of cortical to total bone diameter (CBTg = [M1 – M2]/M1), and CBTavg as the mean combined cortical thickness at both levels (CBTavg = [(M1 – M2) + (M3 – M4)]/2) [16]. All measurements were performed on standardized true anteroposterior radiographs using a calibrated digital imaging workstation (DeepUnity Diagnost 1.1.0.1, Dedalus HealthCare Group).

### Statistical analysis

Data analysis was performed using SPSS version 27 (SPSS Inc., Chicago, IL, USA). Normality was assessed with the Kolmogorov–Smirnov test (*p* > 0.05 indicating normal distribution). Normally distributed variables (age, BMI, AHD, CBTg, CBTavg) were reported as means ± standard deviations; non-normal data as medians and interquartile ranges. Categorical variables were summarized as absolute and relative frequencies. Group differences were evaluated using independent t-tests (normal data), Mann–Whitney U tests (non-normal data), and chi-square tests (categorical variables). Logistic regression was used to determine the association between patient-related and radiographic parameters and the likelihood of cementation. Because of the strong correlation between CBTg and CBTavg, these parameters were entered into separate models to reduce collinearity. The multivariable model included age, sex, CBTavg, CBTg, AHD, and diagnosis. Model performance showed acceptable discrimination (AUC = 0.79). A receiver operating characteristic (ROC) analysis was performed to identify an optimal CBTavg cut-off for predicting cementation. The threshold was determined using the Youden index (J = sensitivity + specificity − 1), and the corresponding AUC and 95% confidence intervals were reported. Odds ratios (ORs) with 95% confidence intervals (CIs) were calculated, and statistical significance was defined as *p* < 0.05. Language editing assistance via AI-based grammar correction tools was used; no content generation occurred.

## Results

### Patient demographics

**Fig. 3:**
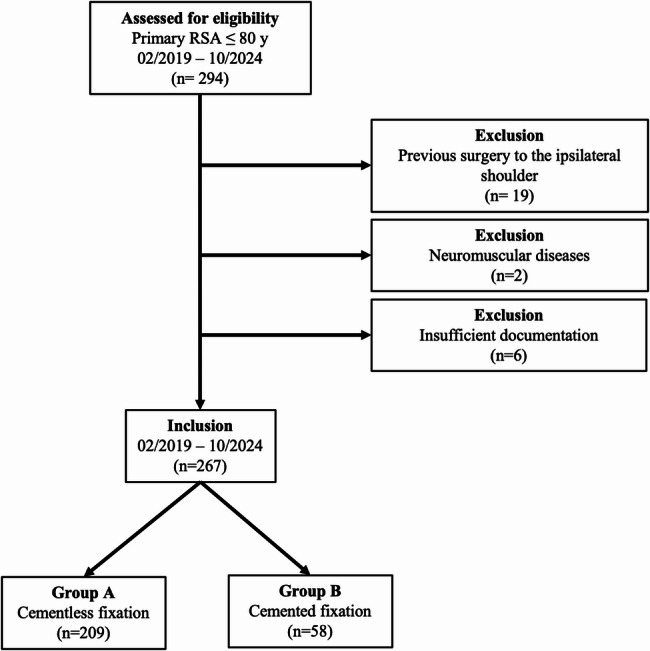
Figure illustrating the patient selection process

A total of 294 patients ≤ 80 years who underwent RSA at our institution between 02/2019 and 10/2024 were screened for inclusion. Of these, patients were excluded due to prior shoulder surgeries (*n* = 19), neuromuscular diseases (*n* = 2), or insufficient documentation (*n* = 6). The final study cohort comprised 267 patients, with 209 patients assigned to the cementless fixation group (Group A) and 58 patients to the cemented fixation group (Group B). Study population is demonstrated in Fig. 3.

**Fig. 4:**
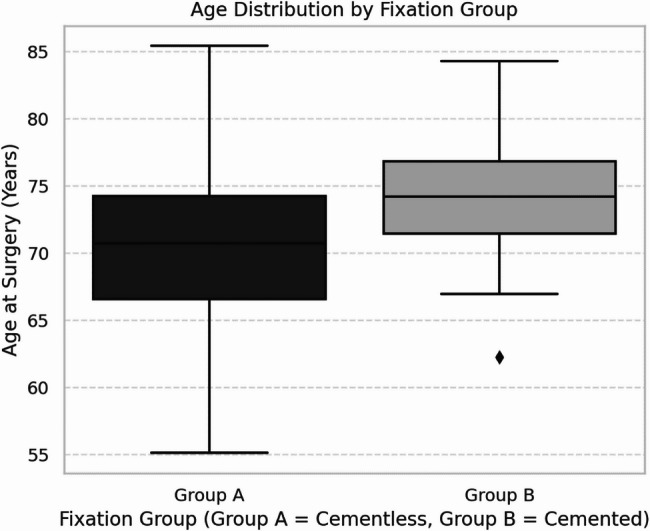
Age distribution across Group A (cementless) and Group B (cemented) fixation in reverse shoulder arthroplasty (RSA). The boxplot illustrates the median age and interquartile range for each group. The mean age in Group A was 70.0 ± 6.1 years, compared to 74.0 ± 4.2 years in Group B

**Fig. 5:**
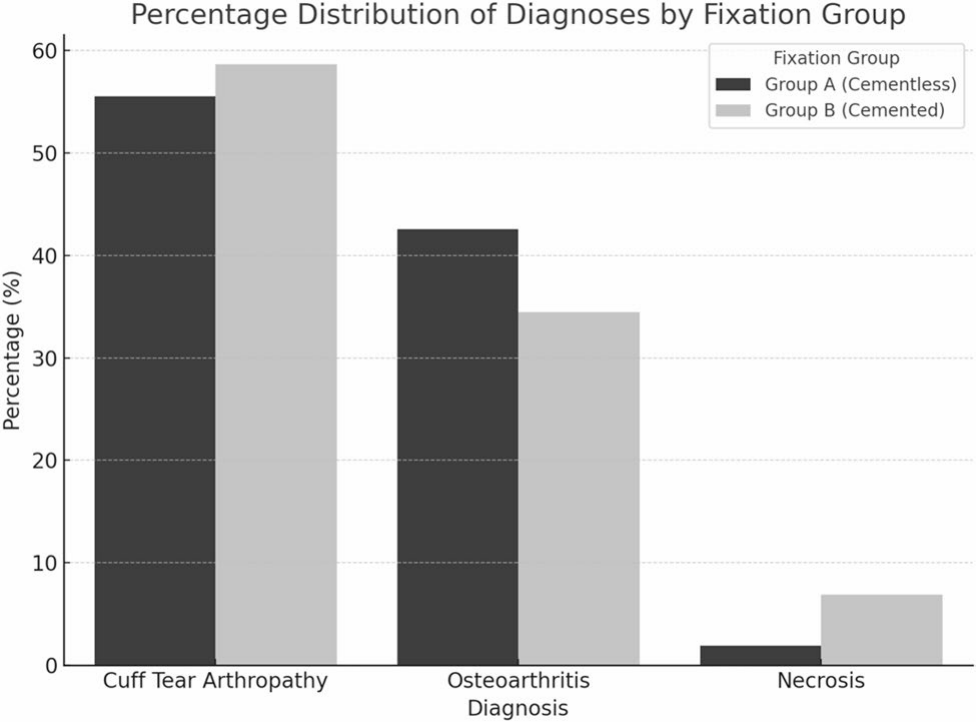
Percentage distribution of diagnoses (cuff tear arthropathy [CTA], osteoarthritis [OA], and HHN) across Group A (cementless) and Group B (cemented) fixation in reverse shoulder arthroplasty (RSA). The bar chart illustrates the predominance of CTA in both groups, a higher proportion of osteoarthritis in Group A, and a slightly increased rate of necrosis in Group B

The mean age at surgery was significantly higher in Group B compared to Group A (74.0 ± 4.2 vs. 70.0 ± 6.1 years; *p* < 0.05) (Fig. 4), and female sex predominated in the cemented group (81% vs. 61%; *p* < 0.05). BMI did not differ significantly between the groups (29.8 ± 6.5 kg/m^2^ vs. 29.6 ± 5.9 kg/m^2^; *p* = 0.83). No significant differences were found in ASA distribution, surgical side, smoking status, or corticosteroid therapy. The distribution of primary diagnoses was similar between groups, with CTA being the leading indication (56% vs. 59%; *p* = 0.49). End-stage OA with humeral head collapse and secondary necrosis (HHN) was more frequent in the cemented group (7% vs. 2%; *p* < 0.05) (Fig. 5). Secondary comorbidities such as diabetes, rheumatic disease, and malignancy were comparable. However, cardiovascular disease occurred significantly more often in the cemented group (34% vs. 17%; *p* < 0.05). The prevalence of osteoporosis was 4% in the cementless group and 10% in the cemented group (*p* = 0.08), indicating no significant difference between groups. An overview of baseline characteristics is provided in Table 1.

## Radiographic assessment

Both cortical bone thickness parameters were significantly associated with the fixation method. The cortical bone thickness gauge (CBTg) was significantly lower in the cemented group (Group B: 0.25 ± 0.06) than in the cementless group (Group A: 0.27 ± 0.06; *p* < 0.05). Similarly, the average cortical bone thickness (CBTavg) was markedly reduced in Group B (5.15 ± 1.23 mm) compared to Group A (6.42 ± 1.43 mm; *p* < 0.05). ROC curve analysis demonstrated that CBTavg had good discriminatory ability for predicting cemented fixation (AUC = 0.76, 95% CI 0.69–0.83; *p* < 0.001). The optimal cut-off value determined by the Youden index was 5.7 mm, providing a sensitivity of 72% and a specificity of 70% for identifying cases requiring cemented fixation. Representative preoperative and postoperative radiographs illustrating cortical measurement methodology and fixation outcomes for both groups are presented in Supplementary Figures S1 and S2. The acromiohumeral distance (AHD) was also significantly smaller in the cemented group (5.22 ± 4.41 mm vs. 7.32 ± 3.79 mm; *p* < 0.05). Other radiographic parameters, including Hamada grade and Walch classification, showed no significant differences between groups (*p* = 0.18 and *p* = 0.23, respectively). Measurement reliability was excellent (interobserver ICC = 0.93; intraobserver ICC = 0.96). Detailed radiographic comparisons are provided in Table 2.

**Table 1:** Comparison of demographic and clinical characteristics between cemented (Group B) and cementless (Group A) fixation in reverse shoulder arthroplasty. Data are presented as mean ± standard deviation (SD) for continuous variables and as absolute numbers with percentages for categorical variables. Significant differences between groups (*p* < 0.05) are marked with an asterisk (*)

	Group A (cementless)	Group B (cemented)	*p*-value
Total number, n	209	58	
Age, y.	70.0 ± 6.1	74.0 ± 4.2	< 0.05*
Sex, m/f	81 (39%)/128 (61%)	11 (19%)/47 (81%)	< 0.05*
BMI, kg/m^2^	29.6 ± 5.9	29.8 ± 6.5	0.83
Diagnosis CTA OA HHN Pre-operated, yes/no	116 (56%) 89 (42%) 4 (2%) 55/154	34 (59%) 20 (34%) 4 (7%) 12/46	0.49 0.16 < 0.05* 0.38
ASA Score			0.10
I	12 (5%)	0 (0%)	
II	112 (54%)	29 (50%)	
III	83 (40%)	28 (48%)	
IV	2 (1%)	1 (2%)	
Secondary diagnosis			
Osteoporosis	9 (4%)	6 (10%)	0.08
Diabetes Cardiovascular	35 (17%) 35 (17%)	13 (22%) 20 (34%)	0.32 < 0.05*
Rheumatic disease Malignant disease	13 (6%) 23 (11%)	0 (0%) 4 (7%)	0.05 0.36
Smoker	25 (12%)	4 (7%)	0.26
Cortisone therapy	9 (4%)	1 (2%)	0.36

**Table 2:** Comparison of preoperative radiographic parameters between cemented (Group B) and cementless (Group A) fixation in reverse shoulder arthroplasty. Data are presented as mean ± standard deviation (SD). Significant differences between groups (*p* < 0.05) are marked with an asterisk (*). Abbreviations: CBTg , cortical bone thickness gauge; CBTavg, average cortical bone thickness; AHD , acromiohumeral distance

	Group A (cementless)	Group B (cemented)	*p*-value
CBTg CBTavg, mm	0.27 ± 0.06 6.42 ± 1.43	0.25 ± 0.06 5.15 ± 1.23	< 0.05* < 0.05*
AHD, mm	7.32 ± 3.79	5.22 ± 4.41	< 0.05*
Hamada Walch	1.9 ± 2.1 1.6 ± 1.8	3.2 ± 1.6 2.11 ± 1.9	0.18 0.23

Given the exploratory nature of this study, no formal correction for multiple testing (e.g., Bonferroni or false discovery rate) was applied. Reported* p*-values should therefore be interpreted as descriptive measures of association rather than confirmatory evidence.

### Multivariate analysis and relative impact of predictors

Multivariate logistic regression analysis identified increasing age (OR: 1.13; 95% CI: 1.07–1.19; *p* < 0.05), female sex (OR: 2.82; 95% CI: 1.43–5.56; *p* < 0.05), and reduced average cortical bone thickness (CBTavg) (OR: 0.59; 95% CI: 0.46–0.75; *p* < 0.05) as independent predictors of cemented humeral fixation (Table 3). End-stage OA with humeral head collapse and secondary necrosis (HHN) demonstrated a higher odds ratio for cemented fixation (OR = 3.41, 95% CI 0.88–13.1), representing a statistically nonsignificant but apparent trend toward an association. Although lower CBTg was associated with cementation (OR: 0.03), the wide confidence interval (95% CI: 0.02–0.49; *p* = 0.18) indicated high variability and limited predictive reliability. Despite showing a significant difference between groups in univariate comparison, the AHD did not remain a significant independent predictor in multivariate analysis (OR: 0.96; 95% CI: 0.88–1.06; *p* = 0.42). The multivariate logistic regression model demonstrated acceptable discriminative ability (AUC = 0.79) and good overall fit (Nagelkerke R^2^ = 0.29).

**Table 3:** Results of multivariate logistic regression analysis assessing predictors of cemented humeral fixation in reverse shoulder arthroplasty (RSA). Significant predictors at the 0.05 level are highlighted in bold. CBTavg , average cortical bone thickness; CBTg, cortical bone thickness gauge; AHD , acromiohumeral distance; CTA , cuff tear arthropathy

	Odds ratio (OR)	95% CI)	*p*-value
Age (year) Female sex	1.13 2.82	1.07–1.19 1.43–5.56	< 0.05* < 0.05*
CBTavg (mm)	0.59	0.46–0.75	< 0.05*
CBTg AHD (mm) Necrosis	0.03 0.96 3.41	0.02–0.49 0.88–1.06 0.88–13.1	0.18 0.42 0.09

**Fig. 6:**
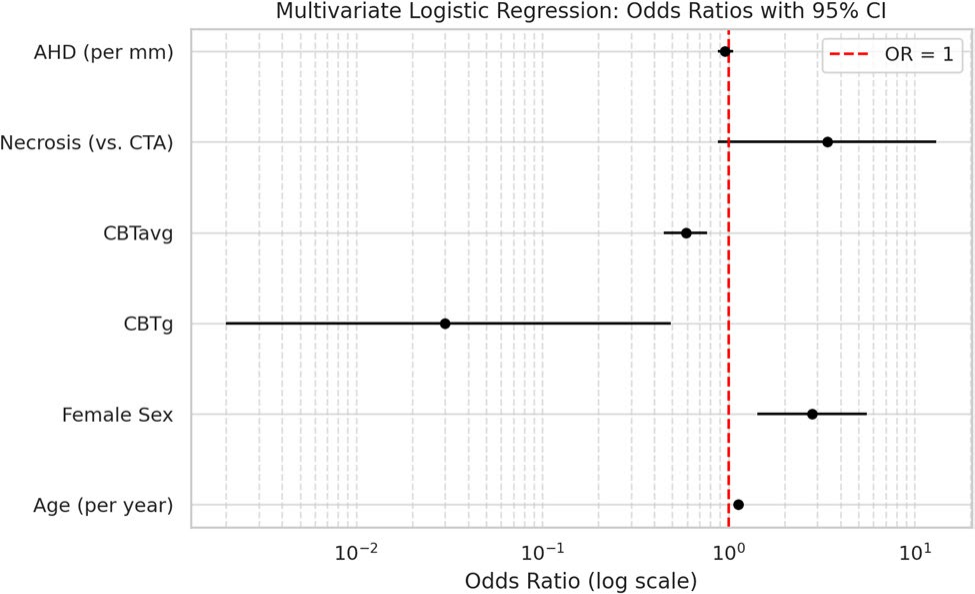
Forest plot of multivariate logistic regression results illustrating odds ratios and 95% confidence intervals for predictors of cemented fixation. Increasing age, female sex, and reduced CBTavg were identified as independent predictors. CBTg and AHD showed no significant predictive value despite trends in univariate analysis. HHN (vs. CTA) showed a statistical trend toward increased odds of cementation

A comparative assessment of these predictors revealed that CBTavg and age had the most consistent and substantial influence on the decision for cementation. Figure 6 illustrates the odds ratios and corresponding 95% confidence intervals for all analyzed predictors.

### Complications

Postoperative complications occurred exclusively in Group A, including one early humeral loosening (1/209, 0.5%) within the first six months after surgery and one periprosthetic fracture (1/209, 0.5%). No complications were reported in Group B during the follow-up period.

## Discussion

This study demonstrates that increasing age, female sex, and reduced average cortical bone thickness (CBTavg) are the strongest independent predictors of cemented humeral fixation in short-stem RSA. Among all radiographic parameters, CBTavg showed the most consistent association with the intraoperative decision, confirming its role as a practical marker of humeral bone quality. While CBTg and AHD differed between groups in univariate analyses, only CBTavg remained independently predictive in multivariate modeling, underscoring its greater robustness for clinical decision-making. The two implant systems used (Ascend Flex and Perform Humeral) share an identical metaphyseal press-fit fixation concept, including preparation with sequential compactors, a coated metaphyseal region, and a polished distal stem. Therefore, implant type was unlikely to have influenced fixation strategy.

Cortical bone thickness is a validated surrogate for local bone mineral density (BMD) across joints [13, 17–19]. In the proximal humerus, Schmidutz et al. demonstrated a strong correlation between CBTg and humeral BMD, confirming the utility of radiographic cortical thickness as a preoperative indicator of local bone strength [13]. Similar associations between cortical thickness and BMD or implant stability have been described in the distal radius, femur, and tibia [17–19]. Our findings extend this concept to RSA: patients with reduced cortical thickness were more likely to undergo cemented fixation, reflecting the technical need for enhanced primary stability in compromised bone. Consistent with these observations, our ROC analysis yielded a preliminary threshold (CBTavg < 5.7 mm) associated with an increased likelihood of cementation (AUC 0.76). While promising, this value requires prospective multicenter validation before being adopted as a standardized criterion. While radiographic cortical thickness serves as a practical surrogate of bone quality, it remains an indirect measure of metaphyseal fixation capacity. To fully understand how cortical morphology influences press-fit mechanics in short-stem RSA, future work will require dedicated biomechanical testing alongside objective intraoperative assessments. Such studies are essential to validate whether radiographic thresholds—such as the proposed CBTavg cutoff—translate into true mechanical stability under controlled loading conditions.

The association between older age and cementation aligns with established evidence showing that reduced bone quality is more frequent in elderly patients [6, 9, 20]. Previous reports highlight advantages of cemented fixation in cases of compromised bone stock, though at the potential cost of higher risks related to cement use [6]. Conversely, cementless fixation continues to advance with improved metaphyseal designs and porous ingrowth surfaces, although radiolucent lines and early migration remain concerns [21]. In our cohort, osteoporosis prevalence was low and comparable in both groups, suggesting age-related cortical thinning may better explain fixation choices than formal osteoporosis diagnoses.

End-stage OA with humeral head collapse and secondary necrosis (HHN) was more frequent in the cemented group and showed a nonsignificant trend toward association in the multivariate analysis. Severe structural degeneration, including metaphyseal sclerosis, likely reduces the potential for reliable press-fit fixation and can therefore shift the intraoperative decision toward cementation [22, 23]. Although the absolute number of HHN cases in our cohort was small and statistical power limited, this pattern is consistent with previous reports indicating that bone-compromised pathologies—such as fractures or post-collapse states—often necessitate cemented implantation to ensure sufficient primary stability [24–26]. Importantly, only patients with OA, CTA, or HHN were included in this study to maintain a homogeneous elective RSA cohort. Proximal humerus fractures and other bone-compromising indications were excluded because they are routinely treated with fracture-specific cemented stems at our institution. Given this focused diagnostic spectrum and the comparable distribution of OA and CTA between groups, it is unlikely that the underlying diagnoses biased the comparison of cemented versus cementless fixation. The small HHN subgroup may have contributed to the higher cementation rate, but its limited size prevents meaningful statistical generalization.

General health status, represented by ASA classification, did not differ significantly between groups, supporting that fixation strategy was driven primarily by local bone factors rather than systemic comorbidity burden. Recent studies underscore the reliability of modern short-stem metaphyseal designs, with favorable early outcomes even in older patients [25, 27], which is consistent with the fixation patterns observed in our cohort. Clinically, the strong association between CBTavg and fixation choice highlights the value of incorporating quantitative bone assessment into preoperative planning. Future randomized comparisons of cemented and uncemented fixation across patient subgroups may help refine these strategies further.

This study has limitations. Its retrospective single-surgeon design may introduce selection bias and circularity because intraoperative assessment of bone quality—both subjective and experience-dependent—directly influenced the fixation method later analyzed against radiographic parameters. Although both stem systems shared equivalent fixation concepts and were used consistently by a highly experienced surgeon, subtle differences in surface characteristics or handling may have influenced press-fit behavior. Group imbalance reflects real-world practice, where cemented fixation is rarely required in patients ≤ 80 years. Long-term radiographic outcomes, including progression of radiolucency or late stem migration, were not assessed. Cortical thickness was measured on radiographs rather than CT or DEXA; however, the applied method is validated, reproducible, and widely used, and remains the standard in many healthcare systems where CT-based diaphyseal imaging is not routinely acquired. Nonetheless, CT-based validation of radiographic cortical thickness measurements is necessary to further substantiate our findings. Finally, projection-related measurement variability cannot be fully excluded, although excellent inter- and intraobserver reliability (ICC 0.93 and 0.96) supports consistency of the radiographic approach.

## Conclusions

In patients ≤ 80 years undergoing short-stem RSA, reduced average cortical bone thickness (CBTavg) emerged as the most robust radiographic predictor for the use of cemented humeral fixation. Increasing age and female sex were also associated with cementation. Preoperative assessment of cortical bone quality using CBTavg may support individualized fixation strategies and improve surgical planning. Future studies should aim to establish objective intraoperative bone quality assessment methods that allow for direct correlation with preoperative CBTavg values and thus enhance decision-making consistency. In particular, defining quantitative CBTavg thresholds that may guide the indication for cemented fixation could improve standardization and reproducibility, potentially supported by quantitative CT analysis or AI-based bone segmentation tools as emerging approaches to increase precision.

## Supplementary Information

Below is the link to the electronic supplementary material.

Supplementary file1

## Data Availability

The datasets generated and analyzed during the current study are stored in the institutional research database of the Department of Orthopaedic Surgery, University of Würzburg, and are available from the corresponding author on reasonable request in accordance with institutional and ethical data protection policies.

## Abbreviations

RSA: Reverse shoulder arthroplasty
CTA: Cuff tear arthropathy
OA: Osteoarthritis
CBTg: Cortical bone thickness gauge
CBTavg: Average cortical bone thickness
AHD: Acromiohumeral distance
ASA: American society of anesthesiologists (Score)
BMD: Bone mineral density
PROM: Passive range of motion
CT: Computed tomography
MIO: Metal increased offset
BIO: Bony increased offset
OR: Odds ratio
CI: Confidence interval
BMI: Body mass index
HHN: End-stage osteoarthritis with humeral head collapse and secondary necrosis
