# Supplementary material for: Lower average cortical bone thickness predicts cemented fixation in short-stem reverse shoulder arthroplasty
Source: J Orthop Surg Res. 2026 Jan 19;21:123. doi: 10.1186/s13018-025-06606-1 (PMC12895917; doi:10.1186/s13018-025-06606-1)
Supplement: Supplementary file 1 — Supplementary file1 [file 13018_2025_6606_MOESM1_ESM.docx]

**Supplementary Material**

**
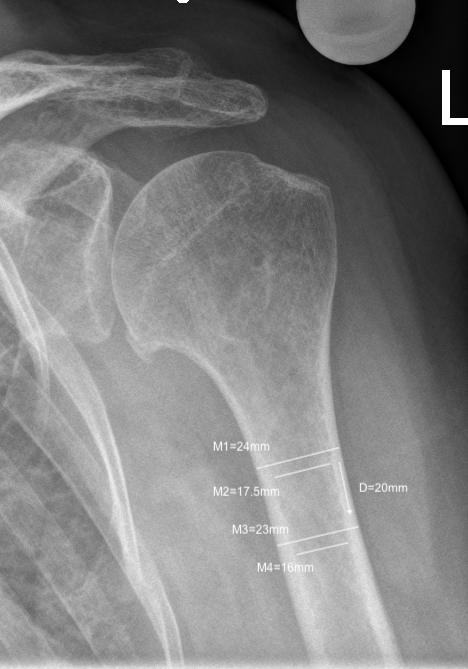

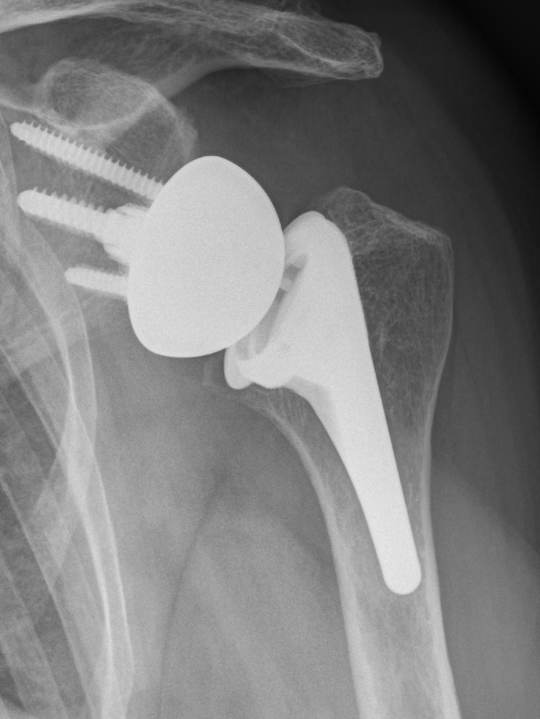
**

**B**

**A**

Figure S1. Representative Case – Cementless Fixation (Group A)

Standardized preoperative true anteroposterior radiograph demonstrating the measurement of cortical bone thickness at two diaphyseal levels (M1/M2 and M3/M4). Measured values: M1 = 24 mm, M2 = 17.5 mm, M3 = 23 mm, M4 = 16 mm. Calculated cortical parameters: CBTg = (M1 – M2) / M1 = 6.5 / 24 = 0.27; CBTavg = [(M1 – M2) + (M3 – M4)] / 2 = (6.5 + 7.0) / 2 = 6.75 mm.

Postoperative radiograph demonstrates a well-aligned, press-fit metaphyseal short stem RSA without evidence of early migration or radiolucency.


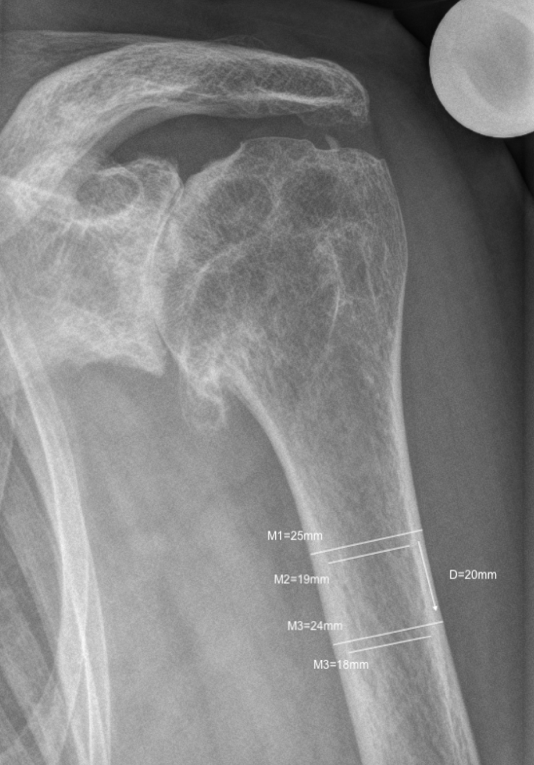

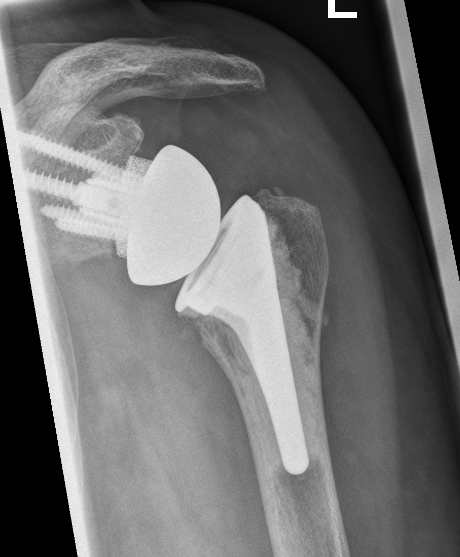


Figure S2. Representative Case – Cemented Fixation (Group B)

Preoperative Standardized preoperative true anteroposterior radiograph with cortical bone thickness measurements at identical diaphyseal levels. Measured values: M1 = 25 mm, M2 = 19 mm, M3 = 24 mm, M4 = 18 mm. Calculated cortical parameters: CBTg = (M1 – M2) / M1 = 6.0 / 25 = 0.24. CBTavg = [(M1 – M2) + (M3 – M4)] / 2 = (6.0 + 6.0) / 2 = 6.0 mm.

Postoperative radiograph shows a cemented short stem RSA with homogeneous cement mantle and appropriate implant positioning.
